# Deconstruction of a memory engram reveals distinct ensembles recruited at learning

**DOI:** 10.1038/s41593-026-02230-2

**Published:** 2026-03-11

**Authors:** Clément Pouget, Flora Morier, Livia Autore, Nadja Treiber, Pablo Fernández García, Nina Mazza, Run Zhang, Isaiah L. Reeves, Stephen M. Winston, Mark A. Brimble, Christina K. Kim, Gisella Vetere

**Affiliations:** 1 Cerebral Codes and Circuits Connectivity Team, Brain Plasticity Laboratory, CNRS UMR 8249, ESPCI Paris, PSL Research University, Paris, France; 2 Biomedical Engineering Graduate Group, University of California, Davis, Davis, CA USA; 3 Department of Surgery, St. Jude Children’s Research Hospital, Memphis, TN USA; 4 Graduate School of Biomedical Sciences, St. Jude Children’s Research Hospital, Memphis, TN USA; 5 Department of Host–Microbe Interactions, St. Jude Children’s Research Hospital, Memphis, TN USA; 6 Center for Neuroscience, University of California, Davis, Davis, CA USA; 7 Department of Neurology, School of Medicine, University of California, Davis, Sacramento, CA USA; 8 Howard Hughes Medical Institute, Princeton, NJ USA; 9 Princeton Neuroscience Institute, Princeton University, Princeton, NJ USA; 10 Omenn Darling Bioengineering Institute, Princeton University, Princeton, NJ USA

**Keywords:** Fear conditioning, Hippocampus

## Abstract

The mechanisms of associative memory formation, including which cells encode a memory and the timing of their engagement, remain poorly understood. By visualizing and tagging cells based on their calcium influx with unparalleled temporal precision, we identified nonoverlapping dorsal CA1 neuronal ensembles that are differentially active during associative fear memory acquisition. We dissected the acquisition experience into periods during which salient stimuli were presented, or certain mouse behaviors occurred, and found that cells associated with specific acquisition periods are sufficient alone to drive memory expression and contribute to fear engram formation. This study delineated the distinct identities of the cell ensembles active during learning and revealed which ones form the core engram and are essential for memory formation and recall.

## Abstract (web summary)

Pouget et al. identified distinct CA1 neuron ensembles active during specific moments of fear learning and uncovered the core engram essential for memory formation.

## Main

Our cognitive system receives a continuous influx of information and processes this data to integrate selected elements into our memory repository. This process involves the activation of specific cell subsets within neuronal networks during memory acquisition and requires the orchestrated reactivation of these subsets later on for memory retrieval^1^. Contextual fear conditioning (FC) in mice, where an association between a neutral stimulus (context, conditioned stimulus) and an aversive one (electric shock, unconditioned stimulus) is established, serves as a common paradigm for studying memory formation. After FC, during subsequent presentations of the conditioned stimulus alone, a conditioned response (freezing behavior) can be observed and measured as an index of fear memory recall^2^. Previous studies have demonstrated that targeted reactivation of cells active at the time of memory encoding across distinct brain areas can artificially induce memory recall^3–6^, indicating the existence of the engram, the physical substrate of a memory trace^7^. The discovery that optogenetic activation of engram cells triggers memory retrieval represented a substantial advancement in our understanding of memory formation. However, what remains unanswered is why certain cells are recruited into the engram and what type of information these cells encode at the time of memory acquisition^8^.

These questions are particularly relevant due to the co-existence of different neurons within brain regions, which are tuned to unique features of an experience. For instance, within the CA1 field of the hippocampus, studies have identified the presence of place cells^9^, shock cells^10^, aversive stimulus-tuned cells^11^ and cells preferentially active during or outside of freezing episodes^12^. Recent research has begun to investigate how these cellular populations are integrated into engrams, in particular those encoding spatial information present at the time of memory acquisition and recall^13^. However, no studies to date have attempted to characterize whether certain cell populations active at distinct periods of encoding have a disproportionate role in memory recall. Exploring this relationship represents a critical step toward understanding how the brain converts specific information from the time of encoding into memories.

A major challenge to addressing this stems from the lack of tools capable of isolating select cells from the broader population active during memory acquisition. Specifically, prior approaches have tagged cells expressing immediate early genes (IEGs), but only over a broad temporal window that can range from several minutes to hours^14^.

Our present work addresses this by using state-of-the-art technology, f-FLiCRE^15^, a new optogenetic tool that uses light and high intracellular calcium for dual-condition tagging. This faster f-hLOV1 variant of FLiCRE, capable of unprecedented temporal resolution, allows for the first time to tag cells active at specific moments of FC, for example, during shock presentation or freezing bouts, and manipulate them in subsequent sessions.

We hypothesize that cells active at crucial periods of memory encoding will be preferentially recruited to the related memory engram.

## Results

### Opto-activation of FC-active dCA1 cells tagged using f-FLiCRE is sufficient for fear memory recall

**Fig. 1:**
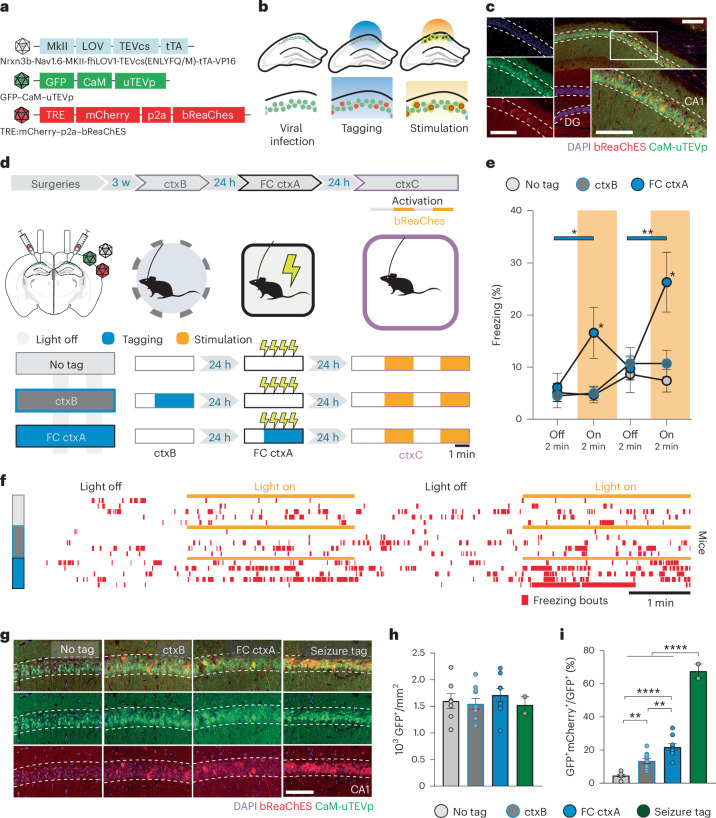
Optogenetic stimulation of f-FLiCRE-tagged dCA1 cells active during FC triggers memory recall. **a**, f-FLiCRE viral constructs with the excitatory opsin bReaChES are injected bilaterally in the dCA1. **b**, Cells expressing f-FLiCRE (green) are tagged using blue light. Tagged cells (red) express mCherry and bReaChES. Later stimulation with yellow light activates previously tagged bReaChES cells. **c**, Example histology of f-FLiCRE-bReaChES infected (GFP^+^, green) and tagged (mCherry^+^, red) cells (DAPI^+^, blue) in the dorsal hippocampus and magnification in CA1. All experimental subjects were processed similarly to confirm viral expression, f-FLiCRE tagging and fiber placement. Scale bars, 100 μm. **d**, Schematics of the experimental protocol (top). Tagging and reactivation timelines for the following three groups: ‘no tag’ (*n* = 7), ‘ctxB’ (*n* = 10) and ‘FC ctxA’ (*n* = 9; bottom). **e**, Comparison of freezing levels across groups in ctxC during light-off (no shading) and light-on epochs (yellow shading). Each data point represents the mean value for one group of mice and is plotted as mean ± s.e.m. across groups. **f**, Freezing timelines of representative individual mice. **g**, Representative images of infection (green) and tagging (red) in the three previously described groups, as well as of a ‘seizure tag’ control (*n* = 2). Scale bar, 100 μm. **h**, Density of GFP^+^ (that is, infected) neurons in the dCA1 layer. **i**, Percentage of tagged cells (GFP^+^mCherry^+^/GFP^+^). Each data point corresponds to the mean value for each individual mouse, while bars represent mean ± s.e.m. across mice. Statistical tests are ordinary one-way or repeated measures (RM) two-way analysis of variance (ANOVA), depending on the case. Statistical differences are depicted with asterisks, with color-coded lines used to show between-epoch comparisons, for consecutive periods only (nonconsecutive significance is not shown). *P < 0.05, **P < 0.01, ****P < 0.001. w, weeks. Source data

**Extended Data Fig. 1:**
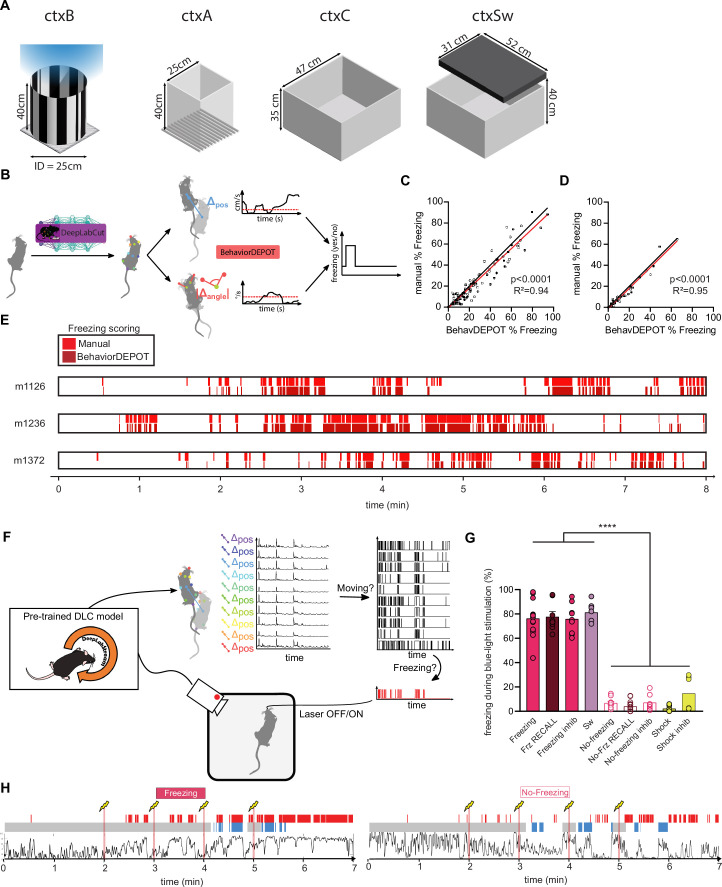
Offline and live automatic freezing scoring methods. **a**, Schematics of the different contexts used. **b**, DeepLabCut and BehaviorDEPOT pipeline for automatic freezing scoring. **c**,**d**, Comparison of automatic and manual freezing scoring with videos split in 20 s bouts (**c**) or in 2-min bouts (**d**) (simple linear regressions). **e**, Example animal freezing scores during ctxC using manual versus BehaviorDEPOT scoring. **f**, DeepLabStream pipeline for freezing/no-freezing detection-dependent closed-loop tagging. **g**, Percentage of freezing during blue light tagging for the different groups. **h**, Example of animals’ post hoc freezing scoring and blue light tagging during FC ctxA. Each data point corresponds to the mean value for an individual animal, while bars represent mean ± s.e.m. across animals. ****p < 0.0001. Source data

Initially, we validated f-FLiCRE efficacy by reproducing a classic gain-of-function experiment to reveal hippocampal engrams in FC^3,4,6^. To this aim, we tagged dorsal CA1 (dCA1) cells with f-FLiCRE (Fig. 1a–c) that were active during FC in context A (ctxA; Extended Data Fig. 1a) for 5 min after the first shock onset. The next day, bReaChES stimulation (in FC-tagged mice) of the tagged cells in a neutral context (context C (ctxC)) triggered freezing, indicating that the activation of FC cells supports aversive memory recall (ctxC; Fig. 1d–f and Extended Data Fig. 1a–e). The increase in freezing was absent in the two control groups that received the same light stimulation in ctxC but without previous tagging (no tag), or tagging for 5 min in a different context in the absence of shocks (context B (ctxB) tag), highlighting the experience-dependent and context-specific nature of our results. Compared to the no tag group, the FC-tag and ctxB-tag groups displayed a significantly higher number of tagged cells (Fig. 1g–i). The FC-tagged cells were also significantly more numerous than ctxB-tagged cells, in line with the observed increase in dCA1 activity associated with high saliency events^16,17^, and suggesting that FC recruits a greater number of neurons above the FLiCRE-tagging threshold. Additionally, the efficacy of f-FLiCRE tagging of dCA1 active cells was validated by quantifying the tagged-to-infected cells ratio during kainic acid-induced seizures (67 ± 5%), known to massively activate neurons. Overall, these results demonstrate that engram tagging based on neuronal calcium influx reproduces previous gain-of-function findings from engram studies using IEGs-tagged cells, and supports f-FLiCRE’s potential as a new, reliable tool for engram tagging with considerably higher temporal resolution.

### dCA1 engram dissection revealed subensembles sufficient to trigger memory recall

**Fig. 2:**
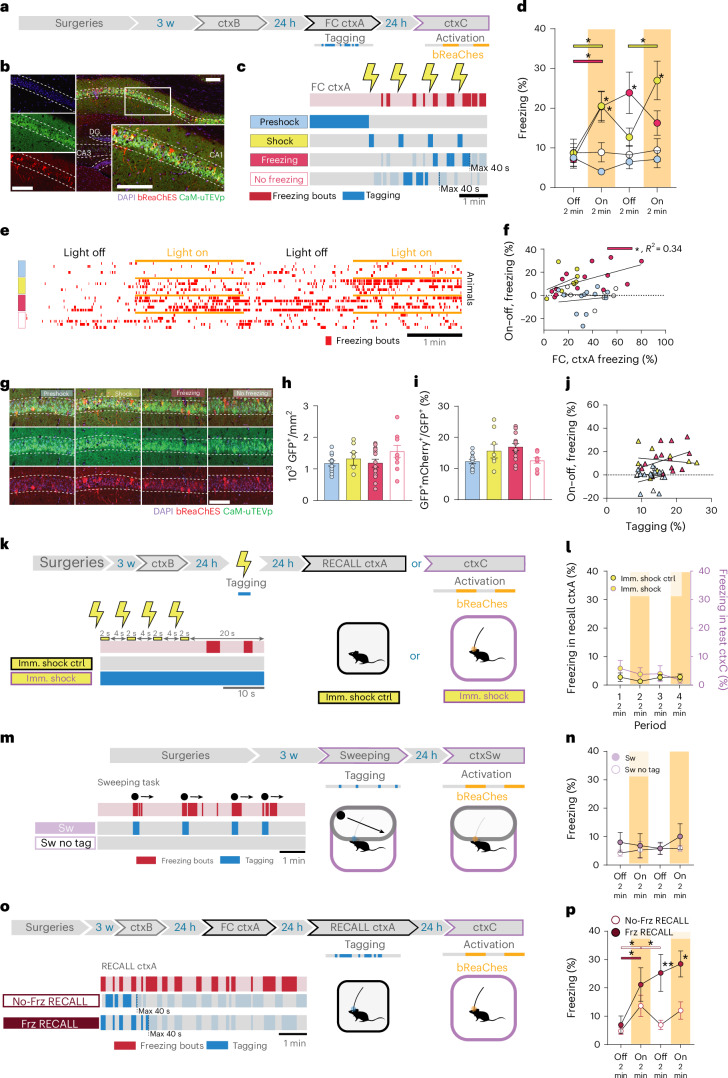
dCA1 cells differentially integrate into the engram depending on when they are active during FC. **a**, Schematic of the experimental protocol—f-FLiCRE viral constructs with the excitatory opsin bReaChES are injected bilaterally into the dCA1; opto-tagging occurs during FC ctxA and opto-reactivation 24 h later during ctxC. **b**, Example histology of f-FLiCRE-bReaChES infected (GFP^+^, green) and tagged (mCherry^+^, red) cells (DAPI^+^, blue) in the dorsal hippocampus and magnification in CA1. Scale bars, 100 μm. **c**, Tagging strategy for ‘preshock’ (*n* = 12), ‘shock’ (*n* = 8), ‘freezing’ (*n* = 14) and ‘no-freezing’ (*n* = 8) groups. **d**, Comparison of freezing levels across groups in ctxC during light-off (no shading) and light-on epochs (yellow shading). Each data point represents the mean value for one group of mice and is plotted as mean ± s.e.m. across groups. **e**, Example individual freezing traces from the four different tagging groups. **f**, Correlation between Δfreezing in ctxC and overall freezing in FC ctxA. **g**, Representative images of infection (green) and tagging (red) in the four groups. Scale bar, 100 μm. **h**, Density of GFP^+^ (that is, infected) neurons in the dCA1 layer. **i**, Percentage of tagged cells (GFP^+^mCherry^+^/GFP^+^). Each data point corresponds to the mean value for each individual animal, while bars represent mean ± s.e.m. across mice. **j**, Correlation between Δfreezing in ctxC and percentage of tagged cells. **k**, Schematics of immediate-shock protocols—opto-tagging occurs during the presentation of immediate shocks; ‘imm. shock ctrl’ (*n* = 10) mice are tested in a recall ctxA session, while ‘imm. shock’ (*n* = 6) mice are opto-reactivated in ctxC. **l**, Freezing-level comparison for the two immediate-shock groups. Left axis is freezing in recall ctxA for ‘imm. shock ctrl’, while the right axis is freezing in test ctxC for ‘imm. shock’ mice. **m**, Schematics of ‘Sw’ (*n* = 7) and ‘Sw no tag’ (*n* = 4) protocol—opto-tagging occurs during sweeping episodes in a modified ctxC and opto-reactivation 24 h later in the same context. **n**, Freezing-level comparison for the two sweeping groups. **o**, Schematics of ‘no-Frz RECALL’ (*n* = 12) and ‘Frz RECALL’ (*n* = 8) groups—opto-tagging occurs during RECALL ctxA 24 h after FC ctxA and opto-reactivation occurs during ctxC, another 24 h later. **p**, Freezing-level comparison for the recall groups. Each data point represents the mean value for one group of mice and is plotted as mean ± s.e.m. across groups. Statistical tests are ordinary one-way or RM two-way ANOVAs, depending on the case. Statistical differences are depicted with asterisks, with color-coded lines used to show between-epoch comparisons, for consecutive periods only (nonconsecutive significance is not shown). *P < 0.05, **P < 0.01. Source data

**Extended Data Fig. 2:**
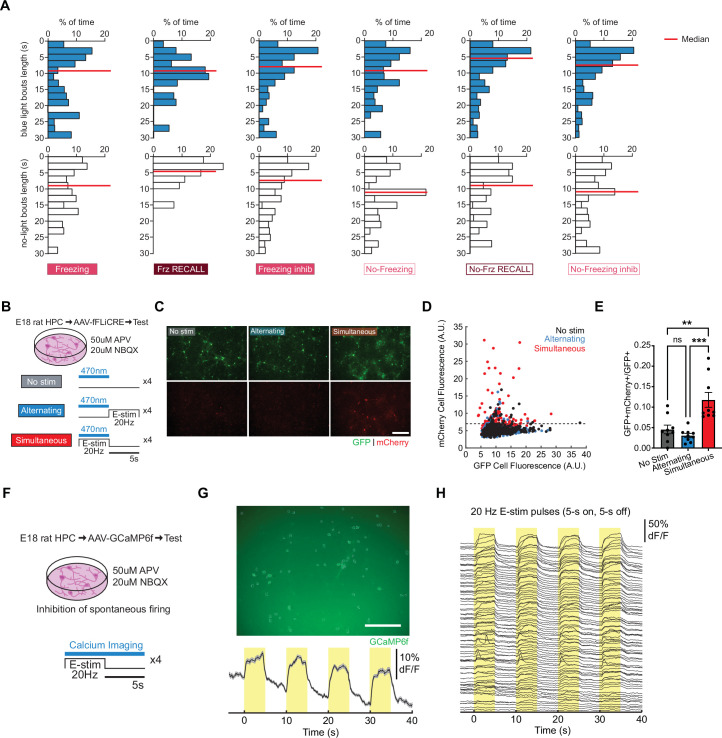
In vitro validation of the new f-FLiCRE tool. **a**, Weighted histograms representing proportionality of blue-light bouts (blue) or no-light bouts (white) for freezing/no-freezing groups. Median in red. **b**, Schematic of the protocol for in vitro f-FLiCRE validation. Neurons expressing f-FLiCRE proteins have their spontaneous firing inhibited with 50 μM APV and 20 μM NBQX, and are stimulated with an electric field at 20 Hz for 5 s at a time. **c**, Example FOVs for all three experimental conditions. Scale bar: 100 μm. **d**, mCherry vs. GFP cell fluorescence from segmented cells. Dotted line represents threshold for positive counting. **e**, Fraction of GFP^+^mCherry^+^/GFP^+^ cells for the three conditions. **f**, Schematic of the protocol for in vitro stimulation/inhibition validation. **g**, Top: FOV of cultured rat hippocampal neurons expressing AAV5-hSyn-GCaMP6f and treated with electric field stimulation for 5 s on, 5 s off, for a total duration of 40 s (5-ms pulse width, 20 Hz pulse frequency). Neurons were treated with 50 μM APV and 20 μM NBQX. Scale bar, 100 μm. Bottom: mean dF/F traces of *n* = 78 neurons detected in the FOV. **h**, Individual traces of the responses of all 78 neuron GCaMP6f to the electric field stimulation. Each data point corresponds to the mean value of an individual FOV, while bars represent mean ± s.e.m. across FOVs. Statistical test is an ordinary one-way ANOVA and Tukey’s multiple comparisons test. **p < 0.01, ***p < 0.001. Source data

To investigate whether cells preferentially active at specific times during memory encoding incorporate different information into the memory engram, we leveraged the full temporal resolution offered by f-FLiCRE. We divided FC into the following four tagging periods: preshock, shock, freezing and no freezing (Fig. 2a–c). To ensure that f-FLiCRE could tag cells active during light-on periods but did not tag cells active during light-off periods, we validated its use by repeatedly delivering bouts of 5-s light on and 5-s light off to cultured hippocampal neurons, matching the median duration of light-on or light-off periods in in vivo ‘freezing’ and ‘no freezing’ experiments (Extended Data Fig. 2a). Neurons either received no stimulation (light only), 20 Hz electric field stimulation during the light-off periods (alternating) or 20 Hz electric field stimulation during the light-on periods (simultaneous; Extended Data Fig. 2b). While ‘light only’ and ‘alternating’ groups showed similar levels of tagging (TRE-mCherry reporter labeled cells), we observed a significant increase in tagged cells only in the ‘simultaneous’ condition (Extended Data Fig. 2c–e). GCaMP6f imaging in separate neurons confirmed that the elevated intracellular calcium levels returned to baseline levels in between bouts of electric field stimulation (Extended Data Fig. 2f–h). These data prove that f-FLiCRE has sufficient temporal resolution and sensitivity to tag even brief bouts of neuronal activity, such as those during ‘freezing’ and ‘no freezing’.

The four subdivisions (‘preshock’, ‘shock’, ‘freezing’ and ‘no freezing’) correspond to crucial periods of FC that together form the experience. During the preshock period, mice explore a new environment and the dCA1 cells process contextual information, before aversive stimulus exposure. Shock delivery constitutes the unconditioned stimulus that triggers a fearful association with the context. After shock delivery, mice engage in different behaviors, including freezing. We measured bouts of freezing as a behavioral readout for the internal fearful state of the animal, which we hypothesized influences memory acquisition. Hence, we tagged cells active during freezing, or outside of freezing bouts, using a closed-loop system that relies on DeepLabStream for online mouse pose estimation and intermittent light delivery^18^ (Extended Data Fig. 1f–h). This choice is further supported by the fact that shock-responding as well as freezing-activated cells have been previously described in the hippocampus^10,12^. To ensure distinct tagging between the ‘shock’ and ‘freezing’ conditions, we controlled that ‘shock-tagging’ epochs included minimal freezing behavior (Extended Data Fig. 1g).

**Extended Data Fig. 3:**
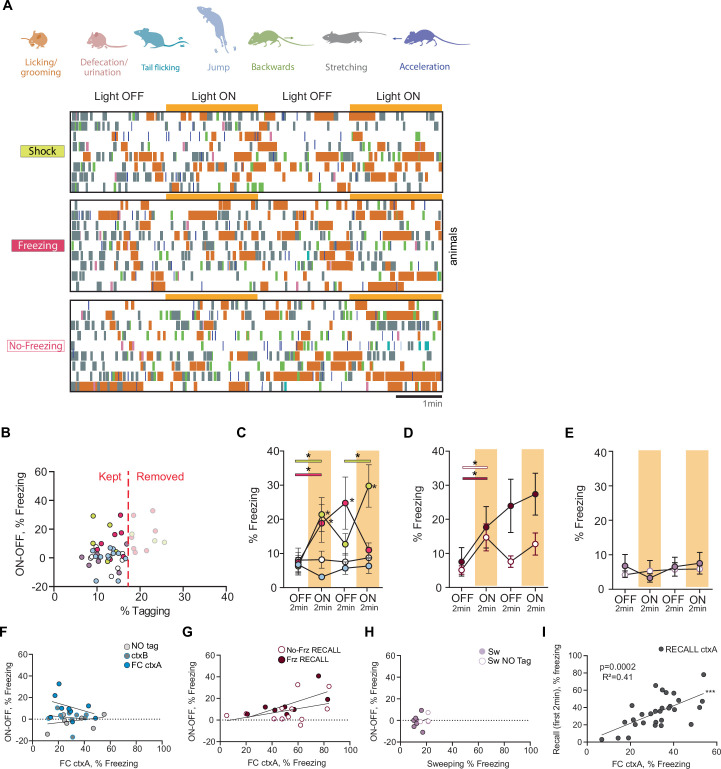
Additional behavioral tracking and counting. **a**, Tracking of various behaviors during ctxC in some example groups and animals (RM one-way ANOVA for each type of behavior is nonsignificant). **b**, Representation of cutoff % tagged value used to compare the behaviors of animals with similar % tagging values. **c**–**e**, Comparison of freezing levels between equivalently-tagged animals during light-off (no shading) and light-on epochs (yellow shading) in (**c**) FC ctxA-tagged animals, (**d**) RECALL ctxA-tagged animals, and (**e**) sweeping-tagged animals. **f**,**g**, Correlation between Δfreezing in ctxC and overall freezing in FC ctxA for (**f**) Fig. 1 groups and (**g**) Fig. 2o,p groups (simple linear regressions). **h**, Correlation between Δfreezing in sweeping-test and overall freezing during sweeping for Fig. 2m,n groups (simple linear regressions). **i**, Correlation between freezing in the first 2 min of recall (that is, before any optogenetic manipulation) and freezing in FC ctxA for all inhibitory groups. Statistical differences between groups are depicted with simple asterisks, while between epochs with color-coded lines, only for consecutive periods (nonconsecutive significance not shown). Each data point corresponds to the mean value for each individual animal, while bars represent mean ± s.e.m. across animals. *p < 0.05, ***p < 0.001. Source data

We assigned different batches of mice to the four mentioned groups and reactivated f-FLiCRE-tagged cells in a neutral context (ctxC) one day after (Fig. 2a–c). The optogenetic reactivation revealed that only ‘shock’-tagged or ‘freezing’-tagged mice exhibited elevated freezing behavior during light-on epochs, while ‘preshock’-tagged or ‘no-freezing’-tagged mice did not (Fig. 2d,e and Supplementary Videos 1–4). Apart from freezing, the different groups exhibited similar behaviors in ctxC (Extended Data Fig. 3a), suggesting that optogenetic stimulation specifically triggers fear memory-like responses. This result suggests that cell populations active at certain distinct encoding periods (‘shock’ and ‘freezing’) have an important role in memory recall, while others do not (‘preshock’ and ‘no freezing’).

The number of infected cells and ratio of tagged-to-infected cells were comparable across all groups (Fig. 2g–i), and ctxC test Δfreezing was not influenced by the tagged-to-infected ratio in any condition (Fig. 2j). Because some individual mice from the ‘shock’-tagged and ‘freezing’-tagged groups displayed high tagged/infected ratios, we confirmed that their exclusion did not impact the behavioral differences observed (Extended Data Fig. 3b–e), further demonstrating the importance of content over quantity of tagged cells.

While ‘shock’ and ‘freezing’ cells can both drive freezing behavior if reactivated, we found significant differences between them. First, FC ‘freezing’-tagged mice did not return to pre-activation freezing levels during the second light-off period, maintaining fear memory recall in the absence of further opto-stimulation, while ‘shock’-tagged mice did. Second, the correlation between the percentage of freezing during FC and the light-driven increase in freezing during test in ctxC (Δfreezing = % freezing on–off) was significant in the ‘freezing’-tagged group (Fig. 2f and Extended Data Fig. 3f–h). This correlation reproduces a natural feature of fear memory recall (Extended Data Fig. 3i) and raises the possibility that reactivation of ‘freezing’-tagged cells may engage dynamics more closely associated with a natural recall than those of ‘shock’-tagged cells, although further investigation is needed.

**Extended Data Fig. 4:**
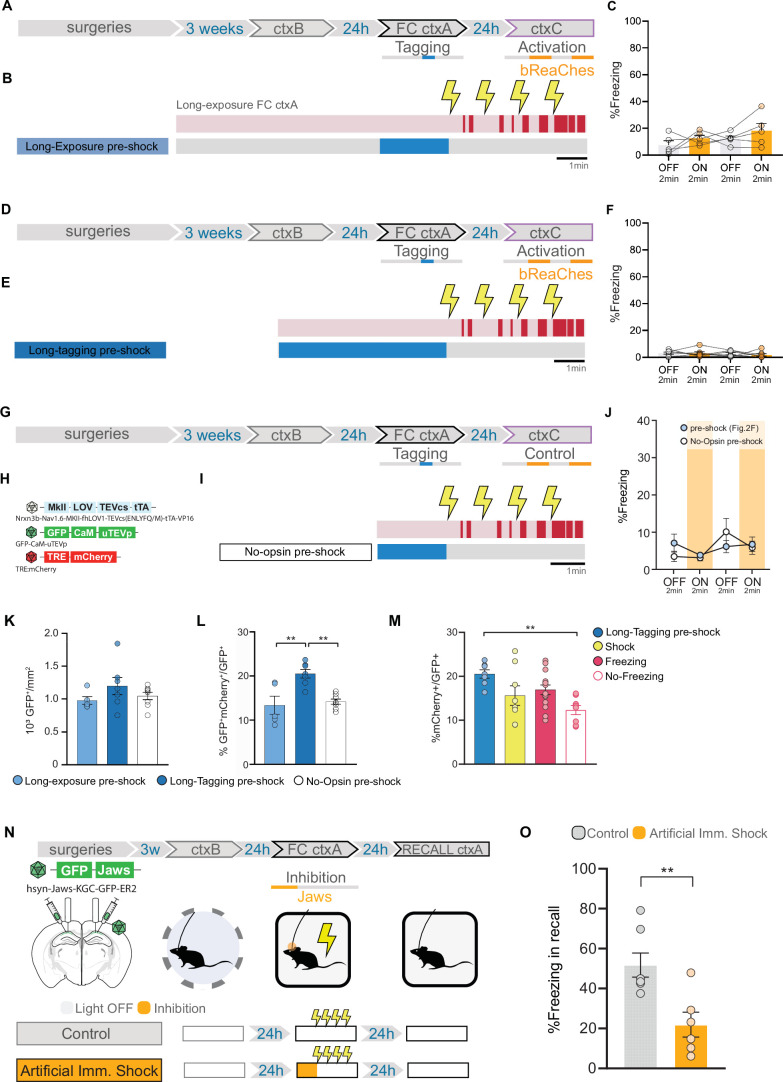
Preshock control groups. **a,b**, Long-exposure preshock experiment protocol (**a**) and tagging timeline (**b**). **c**, Freezing levels of individual ‘long-exposure preshock’ animals during ctxC, outside (gray) and during (yellow) light-on periods. **d**,**e**, Long-tagging preshock experiment protocol (**d**) and tagging timeline (**e**). **f**, Freezing levels of individual ‘long-tagging preshock’ animals during ctxC, outside (gray) and during (yellow) light-on periods. **g**–**i**, No-opsin preshock experiment protocol (**g**), f-FLiCRE no-opsin viral construct (**h**), and tagging timeline (**i**). **j**, Freezing levels of ‘no-opsin preshock’ and ‘preshock’ animals (as already shown in Fig. 2f, here for comparison with no-opsin) during ctxC, outside (gray) and during (yellow) light-on periods. **k**,**l**, Density of GFP^+^ (that is, infected) neurons in the dCA1 layer (**k**) and percentage of tagged cells (GFP^+^mCherry^+^/GFP^+^) in the preshock control groups (**l**). **m**, Percentage of tagged cells (GFP^+^mCherry^+^/GFP^+^) in long-tagging preshock compared to the Fig. 2f groups. **n**, Artificial immediate shock and corresponding control experiment protocols and inhibition timeline. **o**, Freezing level of individual animals during RECALL ctxA, averaged over the whole recall. Each data point corresponds to the mean value for each individual animal, while bars represent mean ± s.e.m. across animals. **p < 0.01. Source data

Our ‘preshock’ results are perhaps surprising considering that the preshock period is essential in creating the unconditioned stimulus association with the context^19^. To eliminate the possibility that contextual information is not sufficiently processed at the time of tagging within these cells, we performed an additional experiment tagging ‘preshock’ cells during long exposure of the context tagging for 5 min, only after an 8-min-long pre-exposure period and controlling with a no-opsin group, and obtained similar results (Extended Data Fig. 4a–m). This experiment confirmed that cells active during FC before unconditioned stimulus delivery are unable to trigger memory recall and may be explained by US-induced remapping of dCA1 neuronal representations^20–22^. Notably, this period is necessary for fear memory formation, as immediate-shock protocols do not create associative contextual memories (Fig. 2k,l), and inhibition of neuronal activity in dCA1 during the preshock period leads to impaired memory recall the following day (Extended Data Fig. 4n,o).

To ensure that the behaviors triggered by the ‘shock’ and ‘freezing’ groups are specifically related to memory, we conducted two additional experiments.

**Extended Data Fig. 5:**
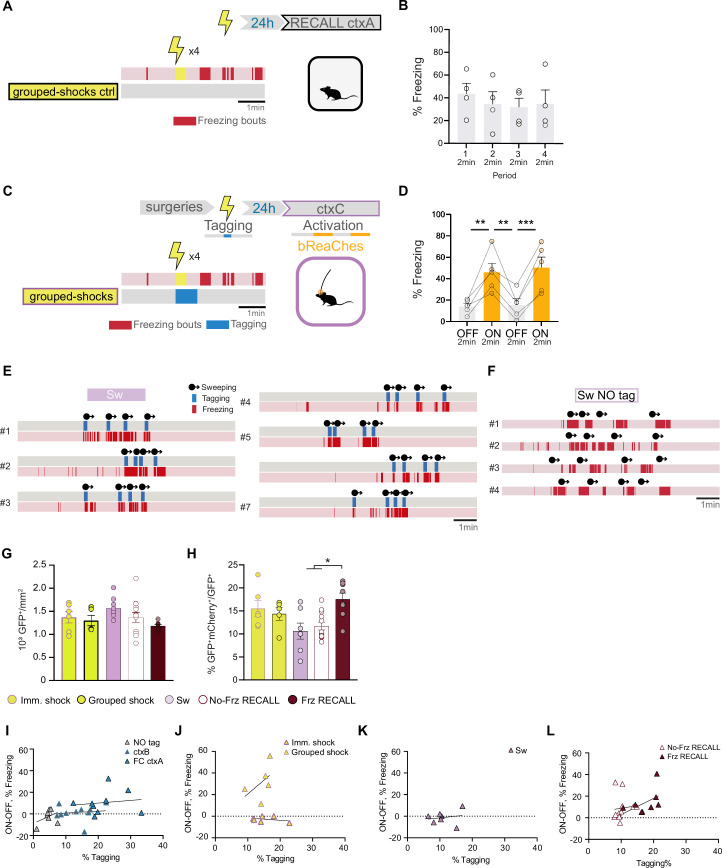
Shock and freezing control groups. **a**, Schematic of grouped-shock ctrl experiment: after training in ctxA with the same shock patterns as immediate shock groups, freezing is tested in a recall ctxA session. **b**, Freezing level in recall ctxA. **c**, Schematic of grouped-shock experiment: after opto-tagging in ctxA with the same shock patterns as immediate shock groups, freezing is tested in an opto-reactivation test ctxC session. **d**, Freezing level in test ctxC. **e**,**f**, Timelines of all ‘Sw’ (**e**) and ‘Sw no tag’ (**f**) animals, displaying sweeping bouts, freezing, and tagging for the former. **g**, Density of GFP^+^ (that is, infected) neurons in the dCA1. **h**, Percentage of tagged cells (GFP^+^mCherry^+^/GFP^+^). **i**, Correlation between Δfreezing in ctxC/sweeping and % of tagged cells for Fig. 1 groups. **j**, Correlation between Δfreezing in ctxC and % of tagged cells for ‘imm. shock’ and ‘grouped shock’ groups. **k**, Correlation between Δfreezing in sweeping-test and % of tagged cells for ‘Sw’ tagged animals. **l**, Correlation between Δfreezing in ctxC/sweeping and % of tagged cells for Fig. 2k,l groups. Each data point corresponds to the mean value for each individual animal, while bars represent mean ± s.e.m. across animals. *p < 0.05, **p < 0.01, ***p < 0.001. Source data

In the first experiment, we tagged ‘shock’-responding cells using an immediate-shock protocol, in which the shock was delivered immediately after placing the mice in ctxA. Under these conditions, mice are unable to form a fear association with the context^19^, as shown by the absence of freezing behavior when re-exposed to the same context the next day (Fig. 2k,l, ‘imm. shock ctrl’). Reactivating ‘shock’-responding cells tagged during immediate-shock protocol did not elicit fear behavior in ctxC the next day, confirming that ‘shock’-responding cells must be linked to an associative fear memory to drive a freezing response. By contrast, consistent with the results for the main ‘shock’ group (Fig. 2d), when the same delay across shocks was applied after 2 min of context exposure, mice successfully formed a memory association (Extended Data Fig. 5a,b), and reactivation of these cells induced freezing behavior in a neutral context (Extended Data Fig. 5c,d).

In the second experiment, we tagged ‘freezing’ cells in a sweeping task^23^ (Extended Data Figs. 1a and 5e,f). Mice freeze as an instinctive response to a perceived overhead threat without an associative memory being formed with the context, as evidenced by the absence of freezing when mice are returned to the same context the next day (Fig. 2m,n, ‘Sw no tag’ control group). Reactivation of these cells in the same context one day later did not elicit freezing behavior (‘Sw’ group), suggesting that the effect observed in the ‘freezing’ group in the FC task is memory-related (Fig. 2d).

As the majority of^3,4,6,24,25^, but not all (see also ref. ^25^), studies targeting the engram have focused on tagging cells during memory acquisition, little is known about the composition of engram cells during memory recall.

To investigate whether specific cells are preferentially involved in memory recall the day after memory formation, we tagged cells active during freezing (‘Frz RECALL’ group) or no-freezing (‘no-Frz RECALL’ group) periods as done previously, but this time during a memory recall session in ctxA (Fig. 2o). Opto-activation of both groups in a neutral context the following day resulted in increased freezing behavior (Fig. 2p). This suggests that, unlike during memory formation, during the memory test, engram cells can be active both during and outside of freezing bouts. This leads to the conclusion that, while only certain acquisition cells are selected to become part of the memory engram, engram cells can be activated at any point during memory recall, whether memory-related behaviors are being expressed or not.

Interestingly, similarly to the FC-‘freezing’-tagged group, recall-‘freezing’-tagged mice did not return to control freezing levels during the second off period, further supporting the idea that different subensembles contribute to the engram in different ways.

Within these additional groups (‘long-exp.’, ‘preshock’, ‘no-Frz RECALL’, ‘Frz RECALL’, ‘Sw’, ‘imm. shock’ and ‘imm. shock ctrl’), some showed differences in expression and tagging (Extended Data Fig. 5g,h); there was no effect of percentage tagging on test Δfreezing in any condition (Extended Data Fig. 5i–l).

### dCA1 engram dissection revealed that the same subensembles are also necessary for memory recall

**Fig. 3:**
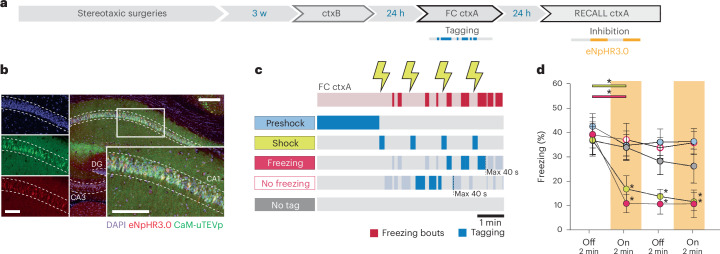
dCA1 cells whose reactivation is sufficient for memory recall are also necessary for memory recall. **a**, Schematic of the experimental protocol—f-FLiCRE viral constructs with inhibitory opsin eNpHR3.0 are injected bilaterally in mouse dCA1; opto-tagging occurs during FC ctxA and opto-inhibition during recall ctxA 24 h later. **b**, Example histology of f-FLiCRE-eNpHR3.0 infected (GFP^+^, green channel) and tagged (mCherry^+^, red channel) cells (DAPI^+^, blue channel) in the dorsal hippocampus and magnification in CA1. All experimental subjects were processed similarly to confirm viral expression, f-FLiCRE tagging and fiber placement. Scale bars, 100 μm. **c**, Schematic of the tagging protocols for ‘preshock’ (*n* = 12), ‘shock’ (*n* = 4), ‘freezing’ (*n* = 6), ‘no-freezing’ (*n* = 7) and ‘no tag’ (*n* = 10) inhibition. **d**, Comparison of freezing levels across groups in recall ctxA during light-off (no shading) and light-on epochs (inhibition, yellow shading). Each data point represents the mean value for one group of mice and is plotted as mean ± s.e.m. across groups. RM two-way ANOVA. Statistical differences are depicted with asterisks, with color-coded lines used to show between-epoch comparisons, for consecutive periods only (nonconsecutive significance is not shown). *P < 0.05. Source data

**Extended Data Fig. 6:**
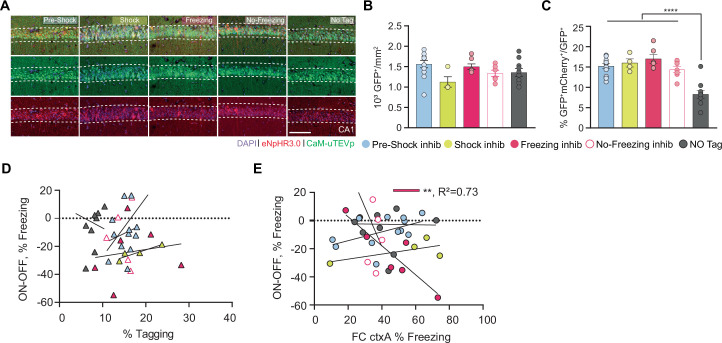
f-FLiCRE-tagging and behavior analysis in inhibitory groups. **a**, Representative images of infection (green) and tagging (red) for inhibitory groups. Scale bar: 100 μm. **b**, Density of GFP^+^ (that is, infected) neurons in the gCA1 for the inhibitory groups. **c**, Percentage of tagged cells (GFP^+^mCherry^+^/GFP^+^). **d**, Correlation between Δfreezing in RECALL ctxA and % of tagged cells for inhibitory groups. **e**, Correlation between Δfreezing in RECALL ctxA and overall freezing in FC ctxA for inhibitory groups. Each data point corresponds to the mean value for an individual animal, while bars represent mean ± s.e.m. across animals. Statistical tests are ordinary one-way or linear regression,s depending on context. **p < 0.01, ****p < 0.0001. Source data

To further demonstrate the distinct integration into the engram of the four subpopulations of cells identified during memory acquisition, we conducted a loss-of-function experiment. We replaced bReaChES with eNpHR3.0, an inhibitory opsin, within the f-FLiCRE system (Fig. 3a). Instead of using the ctxC test session, we introduced a recall session in ctxA, during which we could intermittently inhibit tagged cells. We tagged cells activated during ‘preshock’, ‘shock’, ‘freezing’ or ‘no-freezing’ conditions, replicating the same experimental setup as previously described, and included a ‘no tag’ control group to assess the efficacy of the new reporter protein (Fig. 3b,c). Our findings revealed that only inhibition of the ‘shock’-tagged or ‘freezing’-tagged cells, but not the ‘preshock’, ‘no-freezing’ or ‘no tag’ cells, led to a significant decrease in freezing levels, and that this effect persisted during the second off period in both groups (Fig. 3d). The percentage of infected cells was comparable across groups, percentage of tagged cells was similar for all groups except for the no tag group (Extended Data Fig. 6a–c) and did not correlate with Δfreezing for any group (Extended Data Fig. 6d). These results indicate that the ‘shock’ and ‘freezing’ (but not ‘preshock’ and ‘no-freezing’) subsets of engram cells are not only sufficient (Fig. 2), but also necessary for the behavioral expression of fear memory. Furthermore, paralleling the excitatory results (Fig. 2f), freezing in FC ctxA correlated with Δfreezing only in the ‘freezing’ group (Extended Data Fig. 6e).

### Calcium imaging revealed the presence of nonoverlapping dCA1 neuronal ensembles

**Fig. 4:**
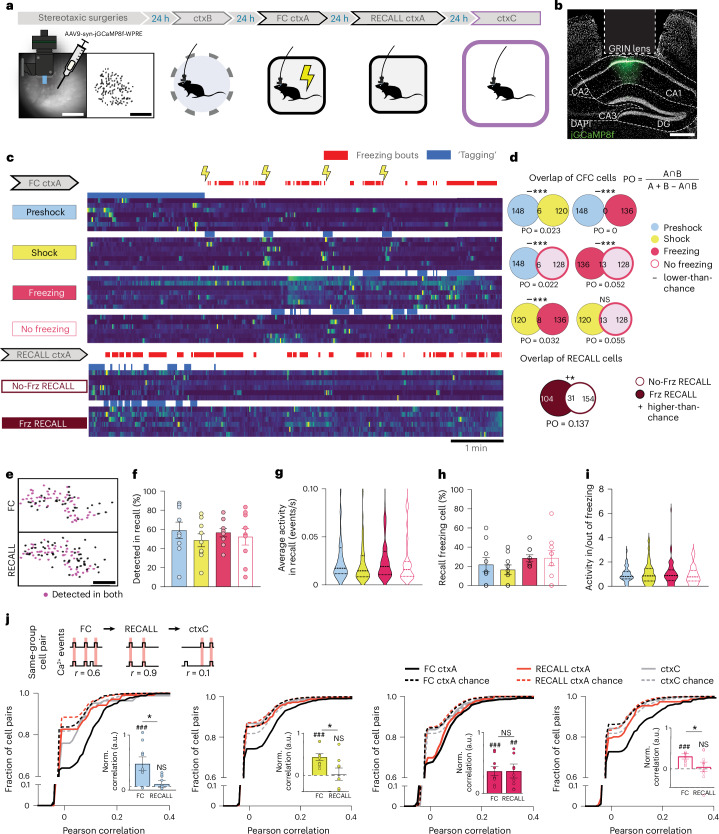
In vivo calcium imaging of FC ctxA-tagged cells. **a**, Schematic of the experimental protocol—jGCaMP8f is injected into dCA1, a relay lens is placed on top of it and a baseplate is installed to allow imaging using the miniscope. Five weeks later, dCA1 cells are imaged across three experiments. Scale bars, 400 μm. **b**, Example histology of injection and lens placement. DAPI is in white and jGCaMP8f is in green. All experimental subjects (*n* = 9) were processed similarly to confirm viral expression, f-FLiCRE tagging and fiber placement. Scale bar, 400 μm. **c**, Example calcium traces from the four different FC ctxA f-FLiCRE-like cell groups, as well as the two RECALL ctxA ones. Blue represents the periods used to determine which cells belong to the corresponding group and would have been tagged using f-FLiCRE. **d**, Overlap across cell groups from the same experiment—top, FC; bottom, RECALL. ‘+’ denotes higher-than-chance overlaps, ‘−’ denotes lower-than-chance overlaps. NS, not significant. Numbers indicate the total number of neurons detected in the cell groups and in their overlap. Proportional overlap (PO) = number of overlap/(number of A + number of B − number of overlap). **e**, Example cell tracking between two experiments using CellReg. Scale bar, 100 μm. **f**, Number of cells per mouse from FC ctxA cell groups detected in RECALL ctxA over chance number by cell group. Each data point corresponds to the mean value for each individual animal while bars represent mean ± s.e.m. across mice. **g**, Average calcium events per second in RECALL ctxA for tracked cells by cell group. **h**, Percentage of FC ctxA groups cells that are freezing cells in test ctxA. Each data point corresponds to the mean value for each individual animal, while bars represent mean ± s.e.m. across mice. **i**, Activity ratios per mouse of FC ctxA groups for cells inside/outside of RECALL ctxA freezing bouts. **j**, Cumulative distributions of same-group cell-pair Pearson correlations in FC ctxA (black lines), RECALL ctxA (red lines) and ctxC (gray lines). Chance-level correlations are shown with dotted lines. Inset graph shows normalized correlations. Each data point corresponds to the mean value for each individual mouse while bars represent mean ± s.e.m. across mice. Statistical tests are comparisons to bootstrapped distributions for overlaps, and ordinary one-way ANOVA otherwise. *P < 0.05, ***P < 0.001, ##*P* < 0.01, ###*P* < 0.001. Source data

**Extended Data Fig. 7:**
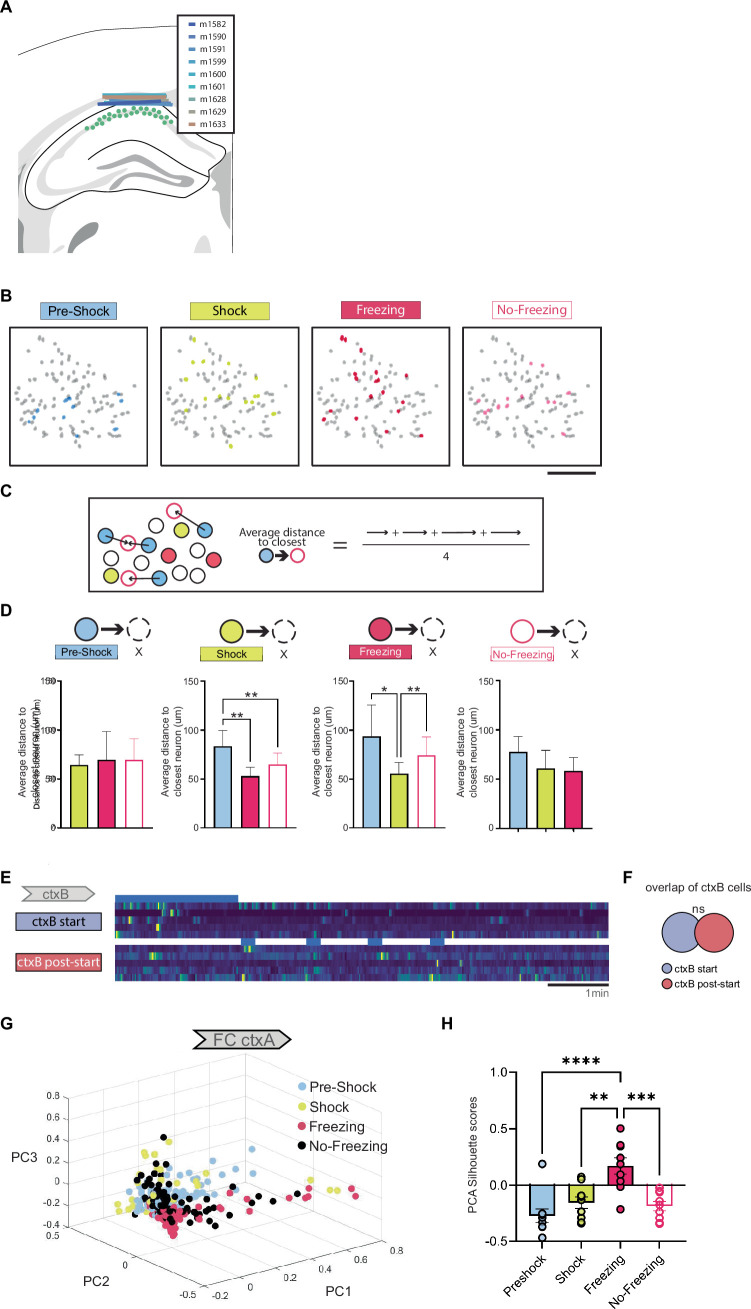
Calcium imaging: validation, overlap statistics, and topological analysis. **a**, Schematic of lens positioning and infection for all 9 animals implanted for calcium imaging experiments. **b**, Representative animal’s miniscope field of view, with the topology of the 4 tracked ‘f-FLiCRE-like’ groups shown. Scale bar: 200 μm. **c**, Computation of average distance to closest neighbor between two cell groups. **d**, Average distance to closest neighbor between all pairs of cell groups (ordinary one-way ANOVA). **e**, Example calcium traces from ctxB. Blue represents the periods used to determine which cells belong to the corresponding group, and would have been tagged using f-FLiCRE. **f**, Overlap between cell groups of ctxB. **g**, Example principal component analysis (PCA) of calcium imaging data during the ‘FC ctxA’ session. Points color-coded according to behavioral periods. **h**, Corresponding silhouette scores for the PCA clusters, that is, different periods. Each data point corresponds to the mean value for an individual animal while bars represent mean ± s.e.m. across animals. *p < 0.05, **p < 0.01, ***p < 0.001, ****p < 0.0001. Source data

To understand the relationship between dCA1 ‘subensembles’ active at the time of encoding and their activity in memory recall, in a separate experiment, we characterized single neuron activity by recording dCA1 calcium dynamics in freely moving mice during ctxB, FC ctxA, recall in ctxA and in ctxC (Fig. 4a,b and Extended Data Fig. 7a). We assigned the cells detected to groups according to their activity in FC ctxA. We then tailored our method to best match f-FLiCRE tagging, as we aimed to study the activity of the same cells that would have been tagged in our optogenetic experiments (Fig. 4c). We first analyzed the overlaps between pairs of subpopulations (that is, the proportion of cells classified as part of both). At encoding, every pair of f-FLiCRE-like cell groups showed significantly less overlap than chance, except for the shock group, that showed chance-level overlap with the freezing and nonfreezing groups (Fig. 4d). This indicates that the four populations are largely separate from one another, despite the greater observed topographic proximity between ‘shock’ and ‘freezing’ cells relative to the other groups (Extended Data Fig. 7b–d). In a nonshocked context (ctxB), the overlap was nonsignificant across cells active in the first 2 min or later on (Extended Data Fig. 7e,f), demonstrating that the significant nonoverlap between ‘preshock’ and other populations was not due to FC-independent intra-experiment changes in the activity. We also analyzed f-FLiCRE-like groups of cells active at recall during freezing or no freezing (‘Frz RECALL’ and ‘no-Frz RECALL’ cells) and found that these two subpopulations showed higher-than-chance overlap levels (Fig. 4c,d).

**Extended Data Fig. 8:**
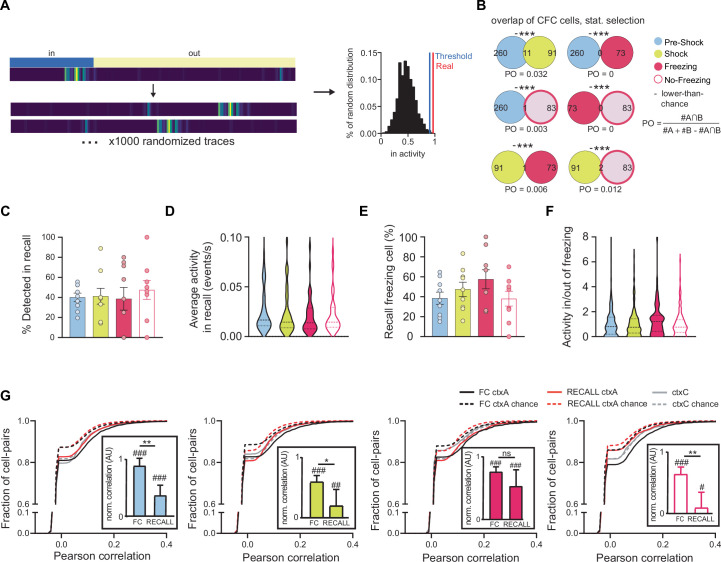
Calcium imaging: analysis with statistical criteria. **a**, Schematic of method for sorting cells into the ‘preshock’, ‘shock’, ‘freezing’, and ‘no-freezing’ groups using a statistical criterion (example shown for preshock). **b**, Overlap between cell groups during FC from the same experiment. + denotes higher-than-chance overlaps, − lower-than-chance overlaps. Numbers indicate the total number of neurons detected in the cell groups and in their overlap. PO: proportional overlap; # overlap / (#A + #B – #overlap). **c**, Number of cells per animal from FC ctxA cell groups detected in RECALL ctxA over chance number by cell group. **d**, Average calcium events per second in RECALL ctxA for tracked cells by cell group. **e**, Percentage of FC ctxA groups cells that are freezing cells in Test ctxA. **f**, Activity ratios per animal of FC ctxA groups for cells inside/outside of test ctxA freezing bouts. **g**, Cumulative distributions of same-group cell-pair Pearson correlations in FC ctxA (black lines), RECALL ctxA (red lines) and ctxC (gray lines). Chance-level correlations are shown with dotted lines. Statistical tests are comparisons to bootstrapped distributions for statistical selection and for overlaps, and ordinary one-way ANOVA otherwise. *p < 0.05, **p < 0.01, ***p < 0.001, #p < 0.05, ##p < 0.01, ###p < 0.001. Source data

We then tracked the activity of the four subpopulations detected in FC ctxA during RECALL ctxA and ctxC (Fig. 4e), hypothesizing that ‘shock’ and ‘freezing’ cells would be preferentially reactivated. However, in RECALL ctxA, we found no differences between any of the f-FLiCRE-like groups in terms of proportion of tracked cells, overall activity, activity during freezing bouts or proportion that were also ‘Frz RECALL’ cells (Fig. 4f–i). These analyses show that neuronal reactivation during memory recall is uniform across the groups and suggest that dCA1 engram reactivation relies on more complex neuronal dynamics. To reveal these dynamics, we computed the correlations in calcium events during FC ctxA, RECALL ctxA and ctxC among same-group cell pairs (Fig. 4j) and found that, for all groups, same-group cell pairs were highly and significantly correlated during FC ctxA. However, only ‘freezing’ cell pairs maintained a significant correlation in RECALL ctxA, while others did not. In ctxC, correlations were nonsignificant for all groups (Fig. 4j). To verify the validity of these results, we ran all the previous analyses again, but switched the method for defining neurons’ identity to a statistical criterion (Extended Data Fig. 8a; Methods) instead of an activity-based one, and obtained identical or highly similar results (Extended Data Fig. 8b–g). Interestingly, dimensionality reduction and clustering analyses revealed that freezing periods were characterized by a distinct dCA1 activity pattern, separable from all other behavioral periods (Extended Data Fig. 8g).

Overall, these results suggest that, although several distinct ensembles of neurons emerge during memory formation, only the ‘freezing’ cells are reactivated as an ensemble during memory recall.

## Discussion

In this study, we show that discrete subpopulations of CA1 neurons are selectively recruited by specific external stimuli (for example, shock delivery) and internal states (for example, freezing behavior) occurring at defined moments during fear memory acquisition. These subensembles are largely nonoverlapping and differ in behavioral relevance—only shock-responsive and freezing-responsive ensembles are both necessary and sufficient to drive fear memory recall, whereas preshock and nonfreezing populations do not elicit memory recall when reactivated.

Recent technological advancements, including FLiCRE technology (but see also references for Cal-Light and FLARE^26–29^), have paved the way for differentiating between and tagging cell populations with higher temporal resolution. This was unachievable with drug-based and IEG-based engram tagging, considering that both the timing of drug delivery and that of IEG-derived protein production occur at a larger timescale than the acquisition window and that the expression of IEGs changes across brain regions, tasks and moments^8,30,31^. Instead, FLiCRE tagging relies on light delivery and calcium level dependencies. The new f-FLiCRE variant that we validate and use here presents even faster kinetics^15^ (Extended Data Fig. 2), allowing us to target cells active in short, intermittent moments (for example, ‘freezing’ bouts) that require the ability to rapidly switch tagging on and off.

The hippocampus, particularly its CA1, CA3 and DG areas, has been central to engram research^3,4,13,23–26^. In our study, we focused on the hippocampal CA1 field due to its known involvement in both formation and recall of recent contextual fear memories^32^. While CA1 is believed to handle the contextual aspect of the experience, it also conveys valence information. Cells such as place cells, shock cells and freezing cells have been observed in the CA1 field, prompting inquiry into their potential differential engagement in the engram^9–12^.

In this study, we found that active cells tagged during the ‘preshock’ period, when mice are presumably processing the contextual component of the experience, failed to trigger freezing behavior the day after the opto-stimulation, in both short-exposure (Fig. 2d) or long-exposure (Extended Data Fig. 4a–c) context protocols. These results likely stem from experience-driven changes in neuronal activity, previously reported as remapping of place cells after the introduction of a fearful stimulus into a context^20,33^, engagement of distinct ensembles to record places and experiences^13^ or drift in neuronal activity^34,35^. Confirming this hypothesis, calcium imaging showed that ‘preshock’ cells are significantly less active after shock and form a subpopulation distinct from and nonoverlapping with the other groups. This shift in recruited neurons immediately after shock delivery has also been previously observed in the prelimbic cortex^36^, where f-FLiCRE inactivation of ‘postshock’ cells, but not ‘preshock’, impaired memory recall the day after. We also found that optogenetic inactivation of dCA1 during the preshock period impaired memory formation, suggesting that, while this window is required for learning, the active neurons during this phase are not themselves incorporated into the final memory engram. It remains possible, however, that preshock-active neurons have a permissive or modulatory role in shaping the circuit dynamics that enable engram formation, without being themselves recruited into the engram. However, we also showed that, even after shock-driven neuronal reconfiguration, not all cells active (see ‘no-freezing’ group) will be selected for the engram. Reports of artificial memory recall driven by reactivation of hippocampal cells active minutes or hours before FC^27^ are not contradictory with the failure to recall we observed when reactivating ‘preshock’ or ‘no-freezing’ neurons. In fact, putative preallocated neurons could be active at any moment during training, including any of the four periods we used for tagging.

We then showed that both ‘shock’ and ‘freezing’ cells are necessary and sufficient for fear memory recall, leading to the conclusion that, during memory encoding, these two populations are selected to become part of the engram. We observed a topographical proximity between the two (Extended Data Fig. 6) that could reflect their sharing of similar inputs or outputs, an intriguing potential explanation for their recruitment during training^37,38^. One possibility is that, to convey mnemonic content effectively to downstream regions, CA1 engages temporally coordinated ‘packets’ of activity, in which ensembles of sufficient size fire together to reliably influence shared target circuits. Despite this observation, we also showed that differences exist between the two—(1) that only the opto-reactivation of dCA1 ‘freezing’ cells and not ‘shock’ cells led to sustained fear memory retrieval even after optogenetic stimulation had ended (Fig. 2d); (2) that artificial recall in the ‘freezing’ group correlated with fear levels at encoding (Fig. 2f and Extended Data Fig. 5e), a typical characteristic of naturally recalled memories (Extended Data Fig. 3e); (3) that ‘shock’ and ‘freezing’ cells formed highly distinct, nonoverlapping populations (although spatially close; Fig. 4d); and (4) that freezing-related neural activity in dCA1 is distinct from other periods suggests that this behavioral state may engage a unique network configuration potentially important for memory encoding (Extended Data Fig. 7). These differences led to hypothesize that, although both populations are selected in the engram (that is, they are both necessary and sufficient to recall the fear memory), they likely contribute through distinct dynamic mechanisms to reactivate the memory trace. To investigate this, we computed the correlations of calcium traces across cells from the same groups across days. We did indeed observe a strong correlation across ‘freezing’ cells, which persisted during fear recall, suggesting that ‘freezing’ cells, unlike ‘shock’ cells, were being reactivated as ensembles^39,40^.

While optogenetic reactivation of certain neuronal populations can trigger memory recall, our calcium imaging analysis revealed that none of the populations is significantly more active than the others during memory recall under normal conditions. This finding underscores the complexity of neuronal ensemble response patterns, which may rely on dynamics not revealed by single-cell analysis^39,41^. It also highlights the need for a deeper understanding of the dynamics that emerge during prolonged ensemble optogenetic stimulation, especially in light of previous evidence that optogenetically tagged engrams can, through reactivation, eventually reconfigure to adopt endogenous firing patterns^42^.

During FC training, we identified four distinct and nonoverlapping (average ~1%) populations of cells. Out of them, only two (‘shock’ and ‘freezing’) were capable of inducing memory recall upon reactivation. In contrast, RECALL cell populations showed greater overlap (~15%), with all tagged cells being capable of triggering artificial memory recall, showing that a more homogeneous population is engaged at memory recall.

Altogether, our results reveal that engrams are formed by selecting specific subpopulations of cells active at specific moments of acquisition (that is, ‘shocks’ and ‘freezing’). These nonoverlapping populations present distinct recall dynamics that are only revealed at higher levels of analysis.

The traditional conceptualization of the engram is evolving, and a deeper understanding of the complex interplay among neuronal populations is needed to fully unravel the mysteries of memory. Our findings revealed the neuronal components of the fear memory engram and opened new doors for understanding memory engrams and the complex dynamics underlying memory formation and recall.

## Methods

### Animals

All mice used were C57BL/6NRj x 129/SV first-generation hybrids, both male and female, obtained by in-house breeding of C57BL/6NRj and 129/SV (Janvier Labs). Mice were housed in groups of two to four (including postsurgery), on a standard food and water diet (ad libitum), under a 12-h light/12-h dark cycle, at a controlled temperature of 20–24 °C and humidity of 45–65%. Mice were 8–9 weeks old at the time of surgery (12–13 weeks old when perfused). All procedures were performed in accordance with the official European guidelines for the care and use of laboratory animals (86/609/EEC), after the policies of the French Committee of Ethics (decrees 87–848 and 2001–464) and after the approval by the ethical committee (reference, 2023-05).

### f-FLiCRE viral production

f-FLiCRE expression vectors (Addgene) were packaged into adeno-associated virus (AAV) serotype DJ (Cell Biolabs, VPK-400-DJ) at St. Jude Children’s Research Hospital. Adenoviral helper genes were provided using the plasmid pHGTI-adeno1 (ref. ^43^). Plasmids were transfected into Adherent 293T cells (ATCC, CRL-3216), 10 × 15-cm dishes per construct, at a 1:1:1 molar ratio using polyethyleneimine ‘max’ (Polysciences, 24765). Media was changed to serum-free DMEM at 16 h after transfection. Cell supernatants and pellets were collected 72 h after transfection. Cell pellets were lysed by five freeze–thaw cycles, lysate was collected and diluted in serum-free DMEM to a volume of 40 ml and pegylated with 40% PEG8000 (Thermo Fisher Scientific) at a 1:5 overall volume (10 ml PEG). Supernatants were directly pegylated with 40% PEG8000 at a 1:5 overall volume. Pegylated lysates and supernatants were incubated at 4 °C for 2 h and then centrifuged for 30 min at 4 °C (4,000*g*). The PEG-containing pellets were resuspended in 10 mM Tris 10 mM Bis–Tris–Propane (pH 9). The resuspended sample was treated with benzonase (Sigma-Aldrich) for 1 h at 37 °C and loaded onto a two-step (1.5 g ml^−1^:1.3 g ml^−1^) CsCl step gradient^44^ in a thick-wall ultracentrifuge tube (Beckman, 360743). Samples were loaded onto an ultracentrifuge (Sw32Ti rotor) and spun at 24,600 rpm at room temperature for 20 h. The full particle-containing fraction was isolated, loaded onto a dialysis cassette (Thermo Fisher Scientific, 66810), and dialyzed 3× in 1× PBS. The dialyzed virus was collected, filtered through a 0.2 μM filter and concentrated by a 100-kDa filter (Amicon) to a volume of 0.5 ml. AAV vectors were titered by qPCR using serial dilutions of purified virus compared against linearized plasmid standard references. Viruses were aliquoted and stored at −80 °C until use. All four f-FLiCRE plasmids were a gift from Alice Ting and are available on Addgene (plasmids 163031, 163032, 163037 and 158703).

### f-FLiCRE virus preparations

Two different viral preparations were used, depending on which reporter protein was needed (mCherry, and either an excitatory or inhibitory opsin). The excitatory preparation consisted of a 1:1:1 mix of AAV-DJ packaged with:

- Nrxn3b-Nav1.6-MKII-f-hLOV1-TEVcs(ENLYFQ/M)-tTA-VP16
- GFP-CaM-uTEVp
- TRE:mCherry-p2a-bReaChES

The inhibitory preparation consisted of a 1:1:1 mix of AAV-DJ packaged with:

- Nrxn3b-Nav1.6-MKII-f-hLOV1-TEVcs(ENLYFQ/M)-tTA-VP16
- GFP-CaM-uTEVp
- TRE-mCherry-p2a-eNpHR3.0-TS

### f-FLiCRE stereotactic surgeries

For all surgeries, mice were anesthetized with an intraperitoneal (i.p.) injection of ketamine/xylazine (100/10 mg kg^−1^) and received local anesthesia through subcutaneous injection of lurocaïne (4 mg kg^−1^) as well as analgesia through subcutaneous injection of buprenorphine (0.1 mg kg^−1^). After every surgery, mice were rehydrated and received analgesia in the form of subcutaneous injections of both 5% glucose water (150 μl) and meloxicam (20 mg kg^−1^).

### f-FLiCRE viral injection

A glass micropipette pulled from a glass capillary (1.5 mm (OD) × 1.17 mm (ID) × 100 mm (L), GC150T-10, Harvard Apparatus) was attached to a 10-μl Hamilton microsyringe (701 N, Hamilton) filled with water. Virus was loaded into the micropipette, separated from water by an air bubble. A syringe pump (single syringe pump, Fischerbrand) was used to control injection. Mice were injected bilaterally into the dCA1 at coordinates −2 mm anteroposterior (AP), ±1.3 mm mediolateral (ML) and −1.4 mm dorsoventral (DV). The glass micropipette was lowered before injecting 800 nl of one of the two f-FLiCRE viral preparations at 0.1 μl min^−1^. We then waited for 5 min before slowly withdrawing it. The wound was then sutured and the mice were allowed to rest.

### Optic fiber implantation

Optic fibers (200 μm diameter; FP400 ERT, Thorlabs) mounted on ceramic ferrules (CFLC230, Thorlabs) were slowly lowered bilaterally to locations above the injection sites at −2 mm AP, ±1.3 mm ML and −1.15 mm DV. Two screws were attached to the skull beforehand. Finally, dental cement (black Ortho-Jet, LANG) was applied to secure the fibers in place and close the incision.

### f-FLiCRE general experimental timeline

To ensure sufficient viral expression, experiments were initiated 4 weeks after viral injection. Optic fiber implantation was performed in week 3 to allow both sufficient postsurgery recovery time and to limit the amount of time mice lived with the fibers implanted. All experiments were then conducted over 2, 3 or 4 days of the 4th week (depending on the group, one experiment per day). Experiments were conducted during the light phase, typically around 9 a.m. to 12 p.m. Mice were killed by intracardiac perfusion in the afternoon after their last experiment.

### f-FLiCRE in vitro experiments

Embryonic day 18 rat hippocampal neurons (BrainBits) were prepared using the manufacturer’s recommended protocol and provided reagents. Neurons were plated on 35-mm glass-bottom dishes (Cellvis) coated with 0.1 mg ml^−1^ poly-d-lysine (Millipore) dissolved in 1× borate buffer (Thermo Fisher Scientific). Dishes were rinsed with sterile deionized water and dried in a tissue culture hood. At days in vitro (DIV) 5, neurons were infected with either AAV5-hSyn-GCaMP6f (2 μl per dish; Addgene, 100837-AAV5) or AAV2/AAV1–hSyn–FLiCRE viruses (300 μl per dish; in-house crude supernatant AAV). The dishes were wrapped in aluminum foil and placed in the incubator. At DIV 14, neurons were treated under various conditions. Before all experiments, neurons were treated with 50 μM APV and 20 μM NBQX. Electric field stimulation was delivered using a stimulus isolator (WPI) connected to two platinum iridium wires (Alfa Aesar) shaped into rectangles that contacted opposite sides of the dish. Stimulation pulses (5 ms width) were delivered at 20 Hz, using the 10 mA current setting on the stimulus isolator. Electric field stimulation was delivered at 20 Hz for 5 s on, and then 5 s off. Light delivery was performed using a 470-nm LED (Thorlabs) placed directly below the dish (5-mW output power measured at the LED). Light was delivered continuously for 5 s on, and then 5 s off. All timing was controlled using an Arduino Due to send time-locked TTL pulses to the stimulus isolator and LED controller box. All imaging (both time lapse and fixed) was performed using a Keyence BZ-X810 fluorescence microscope. Before imaging f-FLiCRE expression, neurons were fixed using ice-cold methanol. GCaMP/GFP was visualized using a 470/40 nm excitation filter and a 525/50 nm emission filter, and mCherry was visualized using a 545/25 nm excitation filter and a 605/70 nm emission filter. Images were analyzed using CellPose (v2.2.3), and custom scripts in Fiji/ImageJ (v2.9.0) and MATLAB (vR2020b). CellPose was used to identify cellmasks from the green fluorescence channel. Cellmasks were then exported to Fiji, where the mean green fluorescent protein and mean mCherry cell fluorescence were calculated for all images. The area was also calculated for all cells. MATLAB was then used to exclude cells under a certain size threshold, and to calculate the mean number of mCherry^+^GFP^+^ cells in each field of view (FOV). Prism 10 was used for statistical analysis and graph making.

### Contexts for behaviors

Behaviors were conducted in four different settings—ctxA was a 24 cm (W) × 24 cm (L) × 40 cm (H) glass-walled square inside a plain, white-walled, sound-proof conditioning chamber (iMETRONIC) with dim white lighting. The chamber had a metal grid floor for delivering foot shocks. ctxB was a 24-cm (ID) × 40-cm (H) black-walled and white-walled cylinder inside the same conditioning chamber. The chamber was fitted with a plastic, textured floor and lit with dim blue light. ctxC was a white-walled and floored 47 cm (W) × 47 cm (L) × 35 cm (H) arena with dim white lighting. For sweeping experiments, we reused the ctxC chamber and fitted a 24″ LCD screen 40 cm above the floor. The screen displayed a plain white background on low luminosity to match the overall lighting intensity of ctxC. In all contexts, a camera filming at 30 FPS was fitted about 50 cm above the floor to record the mice’s behavior. Because the LCD screen obstructed the field of view during sweeping, the camera was offset by approximately 30° to capture the interior of the chamber. All chambers were wiped with 70% ethanol before animal introduction.

### Behavioral experiments

For ctxB sessions, mice were placed in ctxB for a total of 7 min. For FC ctxA sessions, mice were placed in ctxA for a total of 7 min. During this time, mice received four 0.2 mA, 2 s foot shocks at 2, 3, 4 and 5 min. For fear memory recall, mice were placed in ctxA for a total of 8 min. For ctxC sessions, mice were placed in ctxC for a total of 8 min. For the sweeping task, mice were placed in the sweeping chamber for a total of 8 min. A preliminary study helped in determining the correct sweeping parameters to reproducibly trigger freezing bouts in mice—the dot was a diameter of 2.7 cm and displayed 40 cm above the animal. The dot’s start and end points were randomized, but the dot always moved at 7° s^−1^, traversing the screen in about 10 s. We wrote custom MATLAB code using PsychToolbox to manually trigger sweeps of the dot across the screen. This was done at random intervals, and only when the mouse was standing in the middle of the arena. Mice froze for almost the whole dot sweep. Hence, four 10 s sweeps were displayed, at varying times (sweeps were only triggered when the mouse was alert, crossing the arena’s center). For the sweeping test, mice were placed back in the sweeping chamber for a total of 8 min. For ‘imm. shocks’, mice were placed in ctxA for a total of 40 s. Mice received four 0.2 mA, 2-s foot shocks at 2, 8, 14 and 20 s. For ‘grouped shocks’, mice were placed in ctxA for a total of 5 min. Mice received four 0.2 mA, 2-s foot shocks at 2:00, 2:06, 2:12 and 2:18.

### Excitatory (bReaChES) f-FLiCRE laser stimulation

The optogenetic groups using the mCherry-p2a-bReaChES reporter were f-FLiCRE-tagged using a 473 nm (blue) laser that shone continuously at 5 mW. After 24 h, f-FLiCRE-tagged neurons were reactivated using a 589 nm (yellow) laser shone at 5 mW in 20 ms flashes at 10 Hz.

### Excitatory f-FLiCRE-tagging protocol

‘FC ctxA’ tag mice were tagged during FC, for 5 min after the first shock. ‘ctxB’ tag mice were tagged for the last 5 min of ctxB. ‘No tag’ mice did not receive any blue light. ‘Preshock’, ‘shock’, ‘freezing’ and ‘no-freezing’ tag mice were all tagged during FC—‘preshock’ mice were tagged during the 2 min preceding the first shock. ‘Shock’ mice received 10 s of blue light during and after every shock, for a total of 40 s of tagging. ‘Freezing’ mice received blue light whenever they froze after the third shock, totaling 40 s of blue-light delivery. This start time was used to prevent tagging during periods of uncertainty, when estimation of the mouse position was unreliable, thereby ensuring accurate tagging of freezing behavior only. ‘No-freezing’ mice received blue light whenever they were moving enough to not be considered freezing, starting after the second shock, for a total of 40 s of blue-light delivery. This start time ensured the same 40 s of total tagging could be achieved as for the freezing and shock groups. ‘RECALL Frz’ tag and ‘RECALL no-Frz’ mice were tagged during the memory recall session. These mice received 40 s of blue light during either freezing or no-freezing bouts, respectively, without additional conditions. For ‘sweeping’ tag mice, blue light was delivered during the four 10-s presentations of the sweeping dot, totaling 40 s of tagging. ‘Long-exposure preshock’ mice received 2 min of blue light after 5 min of pre-exposure to ctxA. ‘Long-tagging preshock’ mice were tagged for a full 5 min before the first shock. ‘No-opsin preshock’ mice were tagged the same as ‘preshock’ mice but dCA1 in these mice was injected with a cocktail of virus not expressing the opsin. For ‘imm. shocks’, mice received 40 s of blue light for the entire duration of the FC session. For ‘grouped ghocks’, mice received 40 s of blue light between 2:00 and 2:40.

### Excitatory f-FLiCRE manipulation protocol

Optogenetic reactivation experiments were conducted either in ctxC or in the sweeping chamber (‘Sw’ group). In both cases, mice were placed in the chamber for a total of 8 min, receiving yellow-light stimulation in alternating 2 min bouts (2 min off, 2 min on, 2 min off, 2 min on, 20 ms pulses at 10 Hz).

### Inhibitory (eNpHR3.0) f-FLiCRE laser stimulation

The optogenetic groups using the mCherry-p2a-eNpHR3.0 reporter were f-FLiCRE-tagged using a 473 nm (blue) laser shone continuously at 5 mW. After 24 h, f-FLiCRE-tagged neurons were inhibited using a 589 nm (yellow) laser delivered continuously at 5 mW.

### Inhibitory f-FLiCRE-tagging protocol

‘Preshock’, ‘shock’, ‘freezing’ and ‘no-freezing’ inhibition groups were tagged in the same way as their excitatory counterparts (see above).

### Inhibitory f-FLiCRE manipulation protocol

Optogenetic inhibition experiments were conducted in ctxA, one day after FC (that is, during a fear memory recall session). Mice were placed in the chamber for a total of 8 min, receiving yellow-light stimulation in alternating 2 min bouts (2 min off, 2 min on, 2 min off, 2 min on, continuous).

### Experiment control

Whenever a closed-loop setup was not needed (for example, when tagging shocks, during optogenetic manipulation, etc.), we used Bonsai^45^ to synchronize camera recording, light delivery and shocks in the case of training sessions, through an Arduino Due running the Firmata protocol.

### Closed-loop optogenetics

For behavior-dependent tagging experiments (that is, whenever tagging happened during freezing bouts or outside of freezing bouts), we used a combination of DeepLabStream^18^ and custom Python code to track mice during the experiment with our pretrained DeepLabCut^46^ network. Each frame acquired live by the webcam was analyzed at 15 FPS, and 10 body parts were tracked. Each of these body parts’ speed was computed as the distance the body part moved since the previous frame, divided by 1 of 15 s. For each frame, each body part was considered either mobile or immobile using a threshold of 0.5 cm s^−1^. To ensure that the freezing tag groups contained as much actual freezing as possible, and, conversely, for the no-freezing groups, we used two different thresholds of freezing—for the freezing groups, mice were considered freezing if seven or more body parts were immobile. For the no-freezing groups, mice were considered freezing if six or more body parts were immobile. This labeling was then transmitted through an ‘Arduino Due’ running the Firmata protocol to control the tagging laser accordingly. Both the raw video and live tracking were saved for subsequent analysis.

### Seizure tagging experiment

Seizure-tag mice received an i.p. injection of 20 mg kg^−1^ kainic acid—a glutamate agonist—to induce epileptic seizures as described previously^4^. Mice were placed in an isolated chamber and monitored for seizure levels using the mouse-modified Racine scale^47^. After about 1 h, mice reached stage 5–6 seizures (‘rearing and falling with forelimb clonus’), and blue light was shone continuously for 1 min, five separate times, for a total of 5 min of tagging over about 15 min. Mice were then allowed to recover under close monitoring (seizures decreased in intensity and stopped after 1–2 h) and were killed the next day by intracardiac perfusion (see below).

### Artificial immediate-shock experiment

Mice went through the same surgeries as f-FLiCRE mice, but replacing the f-FLiCRE mix with AAV5-hsyn-Jaws-KGC-GFP-ER2 (Addgene, 65014-AAV5). The dCA1 was then inhibited during the full 2-min preshock period of FC ctxA with a 589 nm laser. The laser was turned on immediately before introducing the animals to the chamber. Memory was then tested in a 8-min long RECALL ctxA session the next day.

### Perfusions and immunohistochemistry

Mice were overdosed with ketamine/xylazine (200/20 mg kg^−1^) and perfused transcardially with 30 ml of 0.9% saline, followed by 30 ml of 4% paraformaldehyde in saline. Brains were carefully extracted and placed in 4% paraformaldehyde at 4 °C for 24 h. They were then transferred to 30% sucrose until they sank to the bottom (1–2 days). Brains were then cut with a cryostat in 50-μm coronal slices. Slices were stained with DAPI (1:10,000 in PBS for 5 min) before being mounted on microscope slides with PermaFluor (Thermo Fisher Scientific).

### Imaging

Brain slices were imaged with a confocal microscope (Nikon A1 or Leica SP8), using three lasers—405 nm (DAPI), 488 nm (eGFP) and 561 nm (mCherry). Laser strength was manually adjusted in between slices and mice to match background levels of fluorescence on all three lasers.

### General f-FLiCRE cell counting

We imaged four to six slices per animal, focusing on slices with the best combination of optrode placement and infection quality. Mice without suitable slices (due to subpar fiber placement, poor infection or both) were excluded from further analyses. To determine the proportion of tagged cells in each group, cells were detected using QuPath’s (v0.4.0)^48^ positive cell detection function. Cell bodies were identified from the green channel (GFP-CaM-uTEVp^+^ cells), and positivity was assessed from the averaged red channel value within each detected cell body (mCherry^+^ cells). The same parameters were used for all slices, except for the threshold, which was manually adjusted for each slice to match the experimenter’s manual counting. Cell counts were then unblinded and pooled across mice.

### Post hoc analysis of freezing

Top-down video recordings of behavior were analyzed using pretrained DeepLabCut networks (ResNet152-based, 1,000 training images, 1 M training iterations) to obtain frame-by-frame tracking of ten body parts (nose, neck, left ear, right ear, left side, right side, middle-back, left hindleg, right handleg, tail base). These tracking data were then input into BehaviorDEPOT^49^ for automatic freezing detection. The ‘freezing_velocity’ classifier was used to identify freezing bouts in the videos, combining a threshold on overall mouse speed and head movement speed with the following parameters:

- Velocity threshold—0.3
- Angle threshold—12
- Window width—32
- Count threshold—10
- Min. duration—0.5 s

This automatic freezing scoring was validated by comparing it to a blinded experimenter’s scoring of five behavior videos (Extended Data Fig. 1c,d).

### In vivo calcium imaging viral preparation

AAV9-packed syn-jGCaMP8f-WPRE was ordered from Addgene, using a plasmid gifted by the GENIE project (Addgene viral prep, 162376-AAV9). The stock viral preparation was diluted 1:1 with saline before surgeries to achieve a titer of approximately 10 (ref. ^12^) viral particles per ml.

### In vivo calcium imaging surgeries

For all surgeries, mice were anesthetized with an i.p. injection of ketamine/xylazine (100/10 mg kg^−1^) and received local anesthesia through subcutaneous injection of lurocaïne (4 mg kg^−1^). Analgesia was provided through subcutaneous injection of buprenorphine (0.1 mg kg^−1^). After each surgery, mice were rehydrated and received further analgesia through subcutaneous injections of 5% glucose water (150 μl) and meloxicam (20 mg kg^−1^).

### GCaMP injection and lens implantation

This surgery was performed following previously described methods^50^. In brief, a 1-mm wide craniotomy was made at −2 mm AP and −1.5 mm ML. The dura was opened, and the cortex was aspirated until crossed fibers were observed (approximately −1.1 mm DV). After aspiration, 1 μl of AAV9-syn-jGCaMP8f-WPRE was injected into the dCA1 at −2 mm AP, −1.3 mm ML and −1.3 mm DV. A GRIN lens (GoFoton 1-mm lens) was then implanted at −2 mm AP, −1.5 mm ML and −1.1 mm DV, and secured with cement and screws.

### Baseplate implantation

The baseplate was implanted while attached to the miniscope to visualize the fluorescence FOV for correct positioning. Mice with discernible vasculature and activity had the baseplate secured to the existing lens cement with superglue and additional black cement. For mice with blurry fluorescence and vasculature, baseplating was attempted again 1 or 2 weeks later. If no fluorescence was observed, these mice were kept as companions and killed at the same time as the experimental mice.

### In vivo calcium imaging experimental timeline

On day 1, miniscope-implanted mice were first imaged in a neutral context (ctxB). On day 2, they underwent FC in ctxA (FC ctxA) and were tested on day 3 (RECALL ctxA). On day 4, they were imaged in another neutral context (ctxC).

### Lens placement and GCaMP infection validation

After completing all imaging sessions, mice were perfused, and brain slices were collected and imaged as previously described (that is, DAPI-stained and imaged with a confocal microscope). For each animal, we verified that dCA1 cells were adequately infected (GCaMP fluorescence can be observed ex vivo using a 488 mm laser). GRIN lens placement was verified by identifying the hole it left in the brain and checking its alignment and distance to the granular layer (optimal placement is ~100 to 200 μm above and aligned with the imaged cells).

### Post hoc analysis of calcium traces

We visualized the activity of *n* = 9 mice, with an average of 90 cells per session (min = 47, max = 172, median = 90, quartile1 = 64, quartile3 = 109). Calcium traces were extracted from saved videos with MIN1PIPE (v3.1)^51^ running in MATLAB (vR2021b). Traces were manually inspected before analysis to remove false positives and duplicates.

### Detection of cell groups in FC

During FC, the analysis of calcium traces was matched to the f-FLiCRE experimental procedure. For any given group (for example, ‘preshock’), calcium traces were averaged during the corresponding period (for example, 2 min of preshock) that would have been the f-FLiCRE-tagging period. Cells were then ranked according to this average, and the distribution obtained. All cells above the mean, as well as one s.d., were designated as belonging to that particular group.

### Principal component analysis

Principal component analysis (PCA) was performed separately for each mouse using calcium activity from all recorded neurons during the ‘FC ctxA’ session. To control for potential drift or systematic changes over the course of the session, we residualized time from the neuronal activity before PCA by fitting a linear model to each PCA separately and using the residuals to reconstruct the adjusted data, thereby removing variance attributable to time. Silhouette scores were then computed from the resulting projections to quantify the separation among predefined behavioral periods (higher silhouette scores indicate greater separation from other clusters; lower scores indicate poorer separation).

### RECALL cells

Similarly to the FC cell groups, the calcium activity of each cell during the recall session was averaged during the freezing periods (‘Frz RECALL’ group) or no-freezing periods (‘no-Frz RECALL’ group) of recall. Cells were ranked from highest to lowest, and cells above the mean as well as one s.d. were selected.

### Cell population overlaps

We computed overlaps between these four cell populations. For statistical analysis, we compared these real overlaps to a bootstrapped distribution of overlaps (that is, chance-level overlap). Cell populations were considered significantly overlapped if their real overlap fell above the 95th percentile of the corresponding bootstrapped distribution. Conversely, they were considered significantly nonoverlapped if their real overlap fell below the 5th percentile of the corresponding bootstrapped distribution. Proportional overlaps were defined as proportional overlaps = number of overlap/(number of A + number of B − number of overlap).

### Cell registration across experiments

Cells were tracked across different experiments using CellReg (v1.5.5)^52^, running in MATLAB (vR2021b). In brief, neuronal footprints from all experiments are obtained from the MIN1PIPE analysis. Experiments are then aligned using translations and rotations. The resulting shifted footprints were compared across sessions using spatial correlation, and a probabilistic model was fitted to these correlations to determine positive and negative matchings. We used the following parameters for all sessions:

- Alignment type—translations and rotations
- Model maximal distance—14 microns
- Initial registration type—spatial correlation
- Initial threshold—0.5
- Registration approach—probabilistic modeling
- Final registration type—spatial correlations
- P_same threshold—0.5

### FC cells in recall

Cells tracked from FC ctxA to recall were sorted into their respective groups from FC—‘preshock’, ‘shock’, ‘freezing’ and ‘no freezing’. Percentages of reactivated cells are expressed as a fraction of FC ctxA cells of said group (for example, 40 preshock cells are detected during FC ctxA, and 10 of them are detected in recall, which would result in 25% detected in recall).

### Overall cell activity

Overall cell activity was computed as the average number of calcium events per second, using 1-s binned, 1-s.d.-thresholded raw calcium traces.

### Binned activity

To compute correlations, calcium traces were binned in 1-s bins and *z* scored to remove any activity below 2 s.d. Peaks were then detected using the MATLAB Findpeaks function to determine the position of calcium ‘events’, and create binned arrays of events (0 for a second without events, 1 for a second with at least one event).

### Correlations

Pairwise Pearson correlation coefficients were computed between the calcium activity traces of all pairs of neurons within each animal. These coefficients were used to generate cumulative distribution plots, which are presented in their original, non-normalized form.

### Random correlations

Random correlations were computed by circularly shifting the event arrays by a random amount 1,000 times and correlating these random event arrays together. Each group’s correlations were then compared to the corresponding random correlations to control for sample size effects in the direct comparisons.

### Normalization for bar graphs

For bar graph summaries of correlations, correlation values were thresholded using a two-s.d. criterion to classify neuron pairs as either ‘correlated’ or ‘not correlated’. This threshold was determined relative to shuffled data, and the percentage of correlated pairs was computed for each animal. This approach provides a single, interpretable value per subject and mitigates potential biases introduced by the high proportion of neuron pairs with zero correlation values, a common feature of calcium imaging data with sparsely nonzero time series. Individual points are mice.

### Topological analysis

After analysis of FC ctxA activity, neurons from the different groups were traced back to their position in the miniscope’s FOV during FC ctxA. To determine the average distance of neurons in group X to the closest neuron in group Y, the distance of each neuron in group X to every neuron in group Y was computed, and only the smallest of these distances was kept. These ‘closest neighbor distances’ were then averaged per animal, and statistical analysis was done by comparing the average values per animal. Please note that the average distance of the closest neighbor X → Y can differ from that of Y → X.

### Detection of cell groups: statistical approach

To validate miniscope results obtained with the ‘activity ranking method’, we applied all the same analyses to cells assigned to groups using a statistical criterion instead. For every group, for every cell, we obtain a random distribution of its calcium trace by applying 1,000 random circular shifts to it. We then compare activity between the ‘in’, that is, putative f-FLiCRE tagging for the corresponding group, and ‘out’ period (everything else), for the real trace and all the random ones. A cell is designated as belonging to the corresponding group if the real trace’s in:out ratio of activity exceeds that of 95% associated random distribution. Subsequent analyses were described above.

### Statistics and reproducibility

Mice were assigned randomly to different experimental groups. Data were included in the analysis only after verification of viral expression, f-FLiCRE tagging and fiber placement. Investigators were blinded to group allocation during experiments and outcome assessment of behavioral performances. To avoid litter bias in mouse experiments, experimental groups were composed of mice from different litters and were randomly distributed.

Statistical analyses were performed using GraphPad Prism and MATLAB. Sample sizes, experimental replicates and statistical tests used for each experiment are reported in source data files and figure legends.

### Reporting summary

Further information on research design is available in the Nature Portfolio Reporting Summary linked to this article.

## Online content

Any methods, additional references, Nature Portfolio reporting summaries, source data, extended data, supplementary information, acknowledgements, peer review information; details of author contributions and competing interests; and statements of data and code availability are available at 10.1038/s41593-026-02230-2.

## Supplementary information

Reporting Summary

Supplementary Video 1 Example ctxC session of ‘preshock’-tagged animal. Excerpt from test session in ctxC, from 1 to 3 min (that is, 1-min light off, 1 min withlight on), sped up four times. Freezing is indicated as scored by BehaviorDEPOT.

Supplementary Video 2 Example ctxC session of ‘shock’-tagged animal. Excerpt from test session in ctxC, from 1 to 3 min (that is, 1-min light off, 1 min with lighton), sped up four times. Freezing is indicated as scored by BehaviorDEPOT.

Supplementary Video 3 Example ctxC session of ‘freezing’-tagged animal. Excerpt from test session in ctxC, from 1 to 3 min (that is, 1-min light off, 1 min withlight on), sped up four times. Freezing is indicated as scored by BehaviorDEPOT.

Supplementary Video 4 Example ctxC session of ‘no-freezing’-tagged animal. Excerpt from test session in ctxC, from 1 to 3 min (that is, 1-min light off, 1 minwith light on), sped up four times. Freezing is indicated as scored by BehaviorDEPOT.

## Source data

Source Data Fig. 1 Statistical source data for Fig. 1 (includes the number of animals (*n*) used in each experiment as well as all statistical details, including tests performed, test statistics, degrees of freedom, exact *P* values and summary outputs).

Source Data Fig. 2 Statistical source data for Fig. 2 (includes the number of animals (*n*) used in each experiment as well as all statistical details, including tests performed, test statistics, degrees of freedom, exact *P* values and summary outputs).

Source Data Fig. 3 Statistical source data for Fig. 3 (includes the number of animals (*n*) used in each experiment as well as all statistical details, including tests performed, test statistics, degrees of freedom, exact *P* values and summary outputs).

Source Data Fig. 4 Statistical source data for Fig. 4 (includes the number of animals (*n*) used in each experiment as well as all statistical details, including tests performed, test statistics, degrees of freedom, exact *P* values and summary outputs).

Source Data Extended Data Fig. 1 Statistical source data for Extended Data Fig. 1 (includes the number of animals (*n*) used in each experiment as well as all statistical details, including tests performed, test statistics, degrees of freedom, exact *P* values and summary outputs).

Source Data Extended Data Fig. 2 Statistical source data for Extended Data Fig. 2 (includes the number of animals (*n*) used in each experiment as well as all statistical details, including tests performed, test statistics, degrees of freedom, exact *P* values and summary outputs).

Source Data Extended Data Fig. 3 Statistical source data for Extended Data Fig. 3 (includes the number of animals (*n*) used in each experiment as well as all statistical details, including tests performed, test statistics, degrees of freedom, exact *P* values and summary outputs).

Source Data Extended Data Fig. 4 Statistical source data for Extended Data Fig. 4 (includes the number of animals (*n*) used in each experiment as well as all statistical details, including tests performed, test statistics, degrees of freedom, exact *P* values and summary outputs).

Source Data Extended Data Fig. 5 Statistical source data for Extended Data Fig. 5 (includes the number of animals (*n*) used in each experiment as well as all statistical details, including tests performed, test statistics, degrees of freedom, exact *P* values and summary outputs).

Source Data Extended Data Fig. 6 Statistical source data for Extended Data Fig. 6 (includes the number of animals (*n*) used in each experiment as well as all statistical details, including tests performed, test statistics, degrees of freedom, exact *P* values and summary outputs).

Source Data Extended Data Fig. 7 Statistical source data for Extended Data Fig. (includes the number of animals (*n*) used in each experiment as well as all statistical details, including tests performed, test statistics, degrees of freedom, exact *P* values and summary outputs).

Source Data Extended Data Fig. 8 Statistical source data for Extended Data Fig. 8 (includes the number of animals (*n*) used in each experiment as well as all statistical details, including tests performed, test statistics, degrees of freedom, exact *P* values and summary outputs).

## Data Availability

All data are available in the main text or the supplementary materials. Source data are provided with this paper.

## Code availability

Codes can be found online on Zenodo (10.5281/zenodo.14276916)^53^.
